# Minor depression in older, long-term unemployed people seeking vocational support

**DOI:** 10.1186/s12888-017-1404-1

**Published:** 2017-07-05

**Authors:** Sabrina Pfeil, Katrin Holtz, Kathrin-Andrea Kopf, Ulrich Hegerl, Christine Rummel-Kluge

**Affiliations:** 1 Department of Psychiatry and Psychotherapy, Klinik und Poliklinik für Psychiatrie und Psychotherapie, University of Leipzig, Germany, Semmelweisstraße 10, 04103 Leipzig, Germany; 2 Depression Research Centre, German DepressionFoundation,, Leipzig, Germany

**Keywords:** Minor depression, Older long-term unemployed, Depressive disorder, Severity, Symptoms, Prevalence

## Abstract

**Background:**

Prevalence rates of minor and major depression vary from 0.7 to 6.8 (minor) and 3.8 to 10.9 (major) for the general population. Twenty-two percent of older, long-term unemployed people suffer from major depression. However, the prevalence rate of minor depression (depression on a subthreshold level with less than 5, but more than 1 depression symptom) in this population is unknown. The first aim of this study is to identify that prevalence rate, because we already know that minor depression increases the risk of developing a major depression and this in turn reduces the chances of reemployment what increases social and individual costs at the same time. The second aim is to find out whether there are symptoms that distinguish the different groups “*no depression”*, “*minor depression”* and “*major depression”* in this population. In contrast to the general population, the most frequent symptoms within major and minor depression in older, long-term unemployed people are unknown so far.

**Methods:**

A total of 234 long-term unemployed people (response rate 59%) were included in a study within a project of the Department of Psychiatry and Psychotherapy at the University of Leipzig and an unemployment agency. Based on the results of the Patient Health Questionnaire, the participants were classified as non depressive, minor depressive or major depressive. Descriptive statistics and chi-square tests were performed to identify whether there are symptoms stated by the participants that are more frequent than others, and if the classified groups differ in this regard.

**Results:**

Fifty percent had no depression, 15.6% had a minor depression and 34.4% were suffering from major depression. Difficulty with concentration is the symptom that differentiated the last two groups the most. Fatigue, depressed mood and anhedonia were the symptoms that distinguished participants with minor depression the most from participants with no depression. Main limitations are: The sample was determined by programme conditions, e.g. not all potentially available people participated. The sample may therefore not be representative for the general long-term unemployed. Due to limited resources the PHQ 9 was used instead of a clinical interview to assess minor and major depression.

**Conclusions:**

Results indicate that minor depression in older, long-term unemployed persons is significant, as, almost 16% of the participants were affected in the study. Especially when fatigue is present for a period of more than 2 weeks, people should be informed about the option to consult a primary care physician or professionals from the unemployment agency in order to prevent the possible onset of major depression.

**Electronic supplementary material:**

The online version of this article (doi:10.1186/s12888-017-1404-1) contains supplementary material, which is available to authorized users.

## Background

Meta-analyses show that unemployment is associated with physical and mental illness, in particular depression (e.g. [18, 22, 36]) and suicide [35]. Chen et al. [11] found in their study that long-term unemployed have more psychiatric symptoms than the short-term unemployed. The reduced activity of the affected persons, what is typical for depression, decreases the chances of reemployment while in turn, symptoms of the individual (lower self-esteem, more pessimistic, more depressive) and social costs increase [47]. Hollederer [26] showed longitudinal that psychosocial stress increases for the long-term unemployed and Gordo [21] found that older persons are suffering more from joblessness than younger people. Moreover, Bühler et al. [9] showed that older, long-term unemployed persons are undertreated and heavily burdened. Within their sample, 70% of the participants were affected by depression (depression, dysthymia or double depression), and 61% of these were without any treatment or disorder-specific treatment. In 2009, Liwowsky and colleagues reported prevalence rates of 38% in a comparable sample for depressive disorders. These and other authors argue that depressive symptoms such as poor self-esteem or lack of motivation are major barriers to getting a job, retaining a job or both [7]. However, depressive disorders can be treated effectively by psychological and pharmacologic therapies [14, 16]. According to the Diagnostic and Statistical Manual of Mental Disorders 5th edition (DSM V; [3]), major depression is associated with heavily impairment and burden. Apart from a full-blown major depressive disorder, there are subjects suffering from depressive symptoms below that clinical threshold, who are not being identified as depressive. So called minor depression, defined as “depressed affect and at least one of the other eight symptoms of a major depressive episode” (DSM V), is a prevalent disorder associated with impairment of quality of life and functional disability [24] or even suicidality [42]. Other terms for this disorder are subsyndromal, subthreshold, or subclinical depression. Rodriguez et al. [38] reviewed literature between 2001 and 2011 and found a wide heterogeneity in definition and diagnostic criteria. The authors concluded that patients falling below the diagnostic threshold suffer from difficulties in functioning, and experience a negative impact on their quality of life. In addition, some researchers argue that major depression’s criteria are questionable, because not all depressed cases in need of treatment are identified (e.g. [4, 28]). Furthermore, Cuijpers and van Straten reported in their meta-analytic review [13] that subjects with minor depression carry a high risk of developing major depressive disorder. Those findings had already been reported in a longitudinal study by Sherbourne et al. [39]. So far, prevalence rates of minor depression are only known for the general population: in 2006, Martin and colleagues reported a rate of 5.4% for minor depression (and 3.8% for major depression). These authors also found the Patient Health Questionnaire [32] being a useful tool to identify major and minor depression in the general population.

A further interesting aspect is that some symptoms of minor depression could be more important than others: Judd and Akiskal [27] found sleep disturbances and fatigue being more frequent than other symptoms within people from the general population suffering from minor depression. However Baumeister and Morar [6] found depressed mood and insomnia/hypersomnia (sleep disturbances) to occur more often than other symptoms within that group. They also found that anhedonia is *the* symptom that distinguishes people with a major depression from people with a minor depression best. If we knew which symptoms in minor depression among the specific group of older, long-term unemployed are particularly frequent, we knew which symptoms we must pay particular attention to. We therefore postulate that more frequent symptoms are more important than less frequent symptoms, to identify people in need.

That is why one of our specific research questions is, which symptoms - people with minor depression suffer from - are more frequent than others within the specific group of older, long-term unemployed. Because so far in the literature it has been shown that in the general population sleep disturbances, fatigue and depressed mood are more frequent than other symptoms in minor depression, but there are no studies examining minor depression and frequent symptoms within older, long-term unemployed. We therefore chose an explorative proceeding because literature is not in consensus so far.

Furthermore we already know that minor depression is a prevalent disorder associated with functional disability, impairment of quality of life and intense healthcare use, and that 22% of older, long-term unemployed people suffer from major depression. But prevalence rates for minor depression in this population are unknown so far. Therefore reporting prevalence rates for minor depression in older, long-term unemployed is the main aim of this study. These findings are important because it is well known that unemployment is associated with mental illness - especially depression - and that minor depression increases the risk of developing a major depression. This in turn reduces the chances of reemployment for the people who suffer from depression. But reemployment not only is a protection factor for health [21], but also is a main aim by politicians and physicians because the reemployment chances decrease with an increasing duration of unemployment [21] and in the same time the social costs increase, too [47]. Beside that the individual costs increase because minor depression is associated with considerable functional morbidity and suicidality.

## Methods

### Subjects

Data were collected within a programme offered by the Department of Psychiatry and Psychotherapy, University of Leipzig, and the unemployment agency since 2011. It was financed by the Federal Ministry of Labour and Social Affairs with the aim to improve the vocational reintegration of long-term unemployed people aged 50 and older. The programme is voluntary and offers psychosocial services like training and counselling. Within the study period of 18 months (Aug 2011–Jan 2013), the programme invited every unemployed person over the age of 50 to participate. Inclusion criteria indicated being 50 years or older and unemployed for at least 12 months. From 396 people within the programme, 234 voluntary participants gave their written informed consent and were included in the study, what corresponds to a response rate of 59%. The Ethics Research Committee of the Faculty of Medicine, University of Leipzig approved the study. Participants first met a clinical expert, and then completed the Patient Health Questionnaire (PHQ, [32]). For the data analysis 16 subjects had to be excluded due to missing data in the PHQ, which left 218 participants who were included in the data analyses. 61% of the sample was female and 39% male. More than half (56.4%) of the participants were divorced or separated, 22% never married, 18.8% married or married again and 2.8% widowed. 12.4% of the participants had a higher educational degree, 25.4% a lower one and 62.2% a middle educational degree. The age ranged between 50 and 62 years (mean = 54) and participants’ unemployment ranged between 12 months and over 20 years.

### Measures

The Patient Health Questionnaire [32] is a multiple choice self-rating tool, which comprises eight psychiatric disorders, four threshold disorders and four below a clinical relevance (minor syndromes). The depression module (PHQ 9) consists of nine items measuring depression according to the DSM IV TR (DSM IV TR, [2]), which are the same as in DSM V (DSM V, [3]). Subjects have to state whether a symptom has affected them *not at all, several days, more than half of the days* or *nearly every day* during the last 2 weeks. Apart from suicidality, a criterion is prevalent if people rate it in one of the last two categories. Suicidality is considered prevalent if *several days* was rated. Depression severity can be measured by a sum score or with a categorical algorithm, based on modified formulations of DSM IV TR criteria, which was used in this study [31]. The Patient Health Questionnaire classifies a *major depression syndrome* as having five or more DSM symptoms and at least one core symptom (A-critera) for 2 weeks or longer. Depressive disorders on a minor level, with the presence of more than one ﻿and﻿ less than five symptoms over a period of 2 weeks or longer, are classified as *other depressive syndrome.* It has a sensitivity of 98%, a specificity of 80% and a positive predictive value of 51% [25]. It is widely accepted as a valid instrument for measure of depression severity [20, 29, 33] and was used in this study because of its proven quality criteria: excellent test-retest reliability, excellent criterion [41] and construct validity [29] and responsiveness [31].

For the analyses, the PHQ 9 was used for identifying subjects with having a major depression, a minor depression or no depression. That classification was used to explore what symptoms people within a group distinguish people from another group best and to report prevalence rates for every single group. A person was classified as having a minor depression by reporting a minimum of two and a maximum of four depressive symptoms, where one of the reported symptoms has to include either depressive mood or loss of interest (one of the A-criteria for depression according to DSM IV/V). On the other hand, a full-blown major depression was defined by reporting five or more symptoms with at least one of the A-criteria. Both categories consider the 2 weeks criterion, by postulating depressive symptoms to be present for 2 weeks or longer. Persons who reported no or one symptom were classified as non depressive. That cut-off point was chosen in accordance to many other studies, as it is a very common way to differentiate minor depression from no depression (summerized and reviewed in the article of Rodriguez et al. [38]). On that basis three groups were definded: a major depression group, a minor depression group and a non depression group. Table 1 summarizes the explanations of major and minor depression in the Patient Health Questionnaire and the DSM criteria.

**Table 1:** Major- and minor depression in PHQ 9 and DSM

	PHQ 9	DSM IV TR	DSM V
	Major depression syndrome	Major depression	Major depression
Major depression	At least 5 symptoms are answered *with more than half of the days* or *nearly every day* ^a^, at least 2 weeks present, 1 core symptom^b^	At least 5 symptoms during the same 2-week period, at least 1 core symptom^b^	At least 5 symptoms during the same 2-week period, at least 1 core symptom^b^
Minor depression	Other depressive syndrome	Depressive disorder not otherwise specified	Depressive episode with insufficient symptoms
	2–4 symptoms are answered with *more than half of the days* or *nearly every day* ^a^, at least 2 weeks present, 1 core symptom^b^	Any depressive disorder that does not meet the criteria for a specific disorder	Depressed affect and at least one other symptom, at least 2 weeks present
Non depression	0–1 symptom is answered with *more than half of the days* or *nearly every day* ^a^	Non depressive	Non depressive

^a^suicidality is also taken into consideration if answered with *several days*

^b^depressed mood and loss of interest or pleasure

### Analyses

Descriptive statistics were used for reporting prevalence rates, and for analyzing which specific depression criteria were most prevalent within the three groups of non depressive subjects, subjects with minor depression and major depression. Furthermore, chi-square tests (Kruskal Wallis tests) were used to compare the groups to identify differences between groups and between symptoms within the groups. At first, all three groups were analyzed with chi-square tests to test if they differ in prevalence of each depression criterion. After that, non depressive subjects and the minor depression group, as well as the minor depression group and the major depression group, were compared in order to identify which groups have what in common, or what groups differ in. Therefore, phi-coefficients were calculated. According to Bortz [8], phi-coefficients are interpreted as *very strong* if they are greater or equal to 0.4, as *strong* if they are greater or equal to 0.3, but smaller than 0.4, and as *moderate* if they are greater or equal to 0.2, but smaller 0.3. Values lower than 0.2 are interpreted as *small* and *very small*. All analyses were performed with SPSS 19.0. Like other authors did before (e.g. [40]), the depression symptom *loss of energy* or *fatigue* is called *fatigue* and for the criterion *loss of interest and pleasure* the term *anhedonia* is used for ease purposes. The dataset is available in Additional file 1.

## Results

**Fig. 1:**
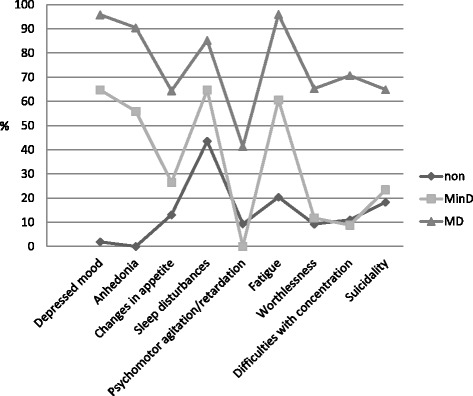
Frequencies of depression symptoms for all 3 groups. Non: no depression group, MinD: minor depression group, MD: major depression group

Fifty percent of older, long-term unemployed people had no depression (0–1 symptoms), 15.6% had a minor depression (2–4 symptoms) and 34.4% were suffering from a major depression (5 or more symptoms). No demographical differences between groups were found (for details see Table 2). Results are shown in Fig. 1. Frequencies for every single symptom in each group are shown in Table 2.

**Table 2:** Demographic characteristics and PHQ 9 diagnosis (*N* = 218)

	Over all	Non depressive (*N* = 109)	MinD (*N* = 34)	MD (*N* = 75)
*Gender (in %)*				
Male	39.0	43.1	35.3	34.7
Female	61.0	56.9	64.7	65.3
Age (mean)	54.0	54.0	54.0	54.0
*Unemployed since (in %)*				
< 5 years	34.0	35.6	35.3	31.0
5–10 years	19.6	16.3	17.6	25.4
10–15 years	17.7	16.3	23.5	16.9
15–20 years	10.5	11.5	11.8	8.5
> 20 years	18.2	20.2	11.8	18.3
*Marital status (in %)*				
Married/married again	18.8	21.1	26.4	12.0
Never married	22.0	22.9	20.6	21.3
Widowed	2.8	3.7	0	2.7
Divorced/seperated	56.4	50.5	52.9	64.0
*Education, highest level (in %)*				
High	12.4	12.1	15.6	11.4
Middle	62.2	55.6	71.9	67.1
Low	25.4	32.3	12.5	21.4

*MinD* minor depression, *MD* major depression

The two A-criteria (depressed mood and anhedonia) are present in about 60% of the minor depression group and in over 90% in the major depression group. Depressed mood and sleep disturbances are existent in 64.7%, respectively, in the minor depression group. Sleep disturbances are present in 43.5% in the non depression group. Even in the non depression group, the suicidal criterion (PHQ 9: recurrent thoughts of death) is the third most mentioned symptom and is present in about 18%.

Chi-square tests revealed significant group differences for every single symptom: every symptom occurs more often in the minor depression group, than in the non depression group and more in the major depression group than in the minor depression group, with the exception of difficulties with concentration and psychomotor agitation/retardation that occur more often in the non depression group than in the minor depression group. Because significant differences were found when all three groups were compared, chi-square pair comparisons were calculated too, which confirm the differences between groups on symptom level. To identify whether subjects with minor depression are more similar to subjects with major depression or to non depressive subjects, phi-coefficients were calculated. Results are shown in Table 3.

**Table 3:** Frequencies of depression symptoms within groups (*N* = 218)

	Frequencies (%)			Statistics			
Depression criterion	Non depressed	MinD	MD	non depr. vs MinD		MinD vs. MD	
				Chi-square	Phi	Chi-square	Phi
Depressed mood	1.9	64.7	95.9	73(1)***	.716	19(1)***	.420
Anhedonia	0	55.9	90.5	69(1)***	.700	17(1)***	.400
Appetite	13.1	26.5	64.4	3(1)	.155	13(1)***	.353
Sleep	43.5	64.7	85.3	5(1)*	.181	6(1)**	.234
Psychomotor	9.3	0	41.3	3(1)	.151	19(1)***	.417
Fatigue	20.4	60.6	96.0	20(1)***	.373	23(1)***	.459
Worthlessness	9.2	11.8	65.3	0(1)	.037	27(1)***	.497
Concentration	11.0	8.8	70.7	0(1)	.030	36(1)***	.573
Suicidality	18.3	23.5	64.9	0(1)	.056	16(1)***	.384

*MinD* minor depression, *MD* major depression

*p < .05, **p < .01, ***p < 0.001

In respect of depressed mood and anhedonia, the minor depression group and the major depression group are more similar to each other. Concerning every other depression criterion the minor depression group has more in common with the non depressive subjects. The greatest difference between minor depression group and major depression group exist for the criterion difficulties with concentration, with a phi-coefficient being very strong (greater than 0.5). For the remaining depression symptoms, the phi-coefficients are higher when the minor depression group and major depression group are compared, with the exception of depressed mood and anhedonia. When the minor depression group was compared with non depressive subjects, no significant differences were found for the symptoms appetite, feelings of worthlessness and guilt, psychomotor agitation/retardation, difficulties with concentration and suicidality, but for fatigue. Moreover fatigue distinguishes people with minor depression best from non depressive subjects. For further information see also Table 3.

## Discussion

### Prevalence rates of minor and major depression among older, long-term unemployed

The main aim of this study was to identify the prevalence rate of minor depression in older, long-term unemployed. Findings of this study suggest that almost 16% report depression symptoms on minor depression level. One third (34.4%) of the sample is burdened by major depression and 50% report no symptom or only one symptom in the last 2 weeks. Martin et al. [33] reported prevalence rates from a representative German community sample with 3.8% for major depression and 5.4% for minor depression, also using the PHQ 9. As can be seen, rates for minor depression and major depression are much higher among the older, long-term unemployed compared to the general population. Liwowsky et al. [30] and Bühler et al. [9] reported rates in older, long-term unemployed about 23% suffering from major depression by using the DIA-X, a fully structured interview based on criteria of the ICD 10 (International Classification of Diseases, Tenth Revision; [46]) and the DSM IV [45]. One possible explanation for the different findings may be the different measurement tools that were used. We believe that our findings are more representative because not only our response rate was higher (59% vs. 42%), but also our study sample larger (*N* = 218 vs. *N* = 44). Compared to Topuzoğlu et al. [43], who studied depression prevalence rates within the general population in Turkey and reported within a subgroup of unemployed participants that 13% suffered from major and 5.3% from minor depression, rates in our study are higher. One possible explanation for the enhanced rate of minor depression in older, long-term unemployed compared to the general population is the fact that minor depression occurs two to three times more frequently especially among an older population, which Meeks et al. [34] showed in their meta-analysis. Moreover, Topuzoğlu and colleagues found being widowed or divorced and being female associated with 2.1-fold higher risk of subthreshold depression (no significant association between marital status and major depression) and unemployment being associated with a three-fold higher risk for major depression. In our study 60% of the participants were female and 59% were widowed or divorced. These findings support our results in high prevalence rates of minor depression in older, long-term unemployed.

What does that mean? So far, we know from findings of Van Zoonen et al. [44] that within their sample of minor depressive people from the general population, only 27% received help and from Topuzoğlu et al. [43] we know that only 24.8% of people with major depression from the general population received minimally adequate treatment. Furthermore we know from Bühler et al. [9] that within their sample of older, long-term unemployed with mental illness, 61% did not receive a disorder specific treatment or any treatment at all, whereas only 9% were treated in accordance to the guidelines. These dramatic findings show the urgent need for action, because reported data let imagine the situation of treatment among older, long-term unemployed with minor depression. Although we know that minor depression is associated with functional disability, impairment of quality of life and with intense healthcare use [24], at this time, no data exist, reporting the situation of treatment within older, long-term unemployed with minor depression.

### Symptoms that are more frequent than others among older, long-term unemployed with minor depression

The second aim of our study was to report whether the kind of present symptoms in the different groups differ in any respect. If we knew which depression symptoms in minor depression are more frequent and thus more relevant than others, subjects suffering from these symptoms could be guided into treatment, in order to minimize the risk for a future major depression. Results of our study show that every single symptom is more frequent in groups of greater severity, what means every single symptom is more frequent in the minor depression group than in the non depression group and every single symptom is more frequent in the major depression group than in the minor depression group, with the exception of difficulties with concentration and psychomotor agitation/retardation. This last point is in contrast to the findings of Baumeister and Morar [6], who reported each symptom being less frequent in minor depression group compared to major depression group, except for sleep disturbances and depressed mood.

In our study sleep disturbances and depressed mood (both 64.7%) were found to be the most frequent symptoms in minor depression group, followed by fatigue (60.6%). These three are followed by anhedonia (55.9%), appetite (26.5%) and suicidality (23.5%). These findings are quite similar to the findings from Judd and Akiskal [27], who reported sleep disturbances (45%), fatigue (42%), suicidality (31%) and depressed mood (30%) as being the most frequent symptoms among subjects with minor depression. Our findings for frequencies of sleep disturbances and fatigue are quite similar to these findings: about two third of the subjects with minor depression in our study suffer from these symptoms. As reported, Judd and Akiskal found sleep disturbances and fatigue being the most frequent symptoms among minor depressive persons. The present study supports these findings, but in contrast to Judd and colleague who analyzed data from a community sample, this study found much higher rates and it found just as frequently depressed mood being present. That is similar to findings from Adams and Moon [1], who reported fatigue and sleep disturbances being about 58% in the older population suffering from minor depression. It could be argued that these findings have nothing to do with depression and are associated with health problems that increase with age, but various authors (e.g. [10, 37]) proved that the PHQ 9 is a reliable and valid instrument for the assessment of depression in elderly (later-life depression). It has been tested and developed for the use with medical patients that are likely (as are elderly) to have high rates of physical symptoms (chronic medical illness).

In addition, what is also interesting about the findings is that sleep disturbances, fatigue and suicidality are present to such a great extent in the non depressive group, too. One possible explanation for this is that the risk for sleep disturbances (and thus fatigue) increases with later life [5]. Gallo et al. [19] also argue that older people have a greater tendency to endorse sleep disturbances and thoughts of death. Furthermore, the suicidality item of PHQ 9 is weighted more sensitive compared to the other items of the scale.

Overall, depressed mood, anhedonia and fatigue not only are the most frequent symptoms being reported in the minor depression group, but also distinguish people with minor depression from people with major depression best. Because fatigue is that important in the minor depression group, it is necessary to get people informed to get in contact with health care services as soon as possible, as symptoms might deteriorate and it would get increasingly more difficult to look for professional help. Future research should take into consideration treatment in older, long-term unemployed suffering from minor depression or major depression, in order to identify reasons for undertreatment. In view of this, Cuijpers et al. [12] suggest that the risk of developing a depression in the future when suffering from minor depression is high and psychological interventions may reduce the incidence of major depression in the future [13].

### Limitations

Our study has five main limitations. First, The PHQ 9 measures depression on a syndrome level. That means other sources of depression, like a physical one, are not taken into consideration. Furthermore it cannot be excluded that the measured depression symptoms within that study are residual symptoms from a recurrent major depression in the past. Second, comorbid disorders were not recorded. Third, a causal link between depression and unemployment cannot be made because of the cross sectional study design. Fourth, the self-reported measurement of all variables could artificially raise associations of depression with all important variables. The prevalence rates reported here may vary from others using different inventories. Fifth, although response rate may be regarded as satisfactory, non-responders might have higher or lower depressive symptomatology than respondents.

However, the prevalence rates of this study emphasize the clinical and public health importance of the topic, especially in older, long-term unemployed people, who are often undertreated and highly burdened, not only from major depression, but also from minor depression. As a result, professionals from the unemployment agency should attract notice to older, long-term unemployed who suffer especially from fatigue. In view of social disability and predictive power of further disability, minor depression needs recognition and sometimes even treatment [15]: counseling like active monitoring, internet based guided self help activities, in special cases treatment with antidepressants [23, 24] or short-term person-centred counselling and low-intensity cognitive behaviour therapy can be reasonable [17].

## Conclusions

The results of the current study indicate that minor depression in older, long-term unemployed persons is significant, as, almost 16% of the participants were affected. Especially when fatigue is present for a period of more than 2 weeks or longer, it should be pointed out that people might consult a primary care physician or professionals from the unemployment agency in order to prevent the possible onset of major depression.

## Additional file

Additional file 1: Dataset of the study. Table with all variables, their labels and their values. (XLS 439 kb)
